# Extraction and Assessment of Features Using Shannon Entropy and Rényi Entropy for Chatter Detection in Micro Milling

**DOI:** 10.3390/mi16020161

**Published:** 2025-01-30

**Authors:** Zehui Zheng, Xiubing Jing, Bowen Song, Xiaofei Song, Yun Chen, Huaizhong Li

**Affiliations:** 1 Key Laboratory of Equipment Design and Manufacturing Technology, Tianjin University, Tianjin 300072, China; 2020201168@tju.edu.cn (Z.Z.); bowensong@tju.edu.cn (B.S.); xiaofeisong@tju.edu.cn (X.S.); 2 Pen-Tung Sah Institute of Micro-Nano Science and Technology, Xiamen University, Xiamen 361102, China; yun.chen@xmu.edu.cn; 3 School of Engineering & Built Environment, Gold Coast Campus, Griffith University, Southport, QLD 4222, Australia

**Keywords:** chatter detection, chatter features, Shannon entropy, Rényi entropy, micro milling, process stability

## Abstract

Chatter is a common phenomenon in micromachining processes that adversely affects machining quality, reduces tool life, and generates excessive noise that contributes to environmental pollution. Therefore, the timely detection of chatter is crucial for sustainable production. This paper presents an investigation on the extraction of two types of features, i.e., probability-related and entropy-related, using Shannon entropy and Rényi entropy algorithms, respectively, for chatter detection in micro milling. First, four chatter features were examined using actual machining tests under stable, weak-chatter, and severe-chatter conditions. Second, the proposed chatter features were systematically assessed by combining the characteristic change rates, threshold intervals, and computation times. The results demonstrated that the proposed features can effectively detect the occurrence of chatters at various severity levels. It was found that the probability-related features exhibit better sensitivity compared to entropy-related features, and the features extracted from Shannon entropy algorithm are more sensitive than the Rényi entropy algorithm.

## 1. Introduction

Micro milling plays an important role in producing micro-devices with complex 3D geometries due to its advantages in high efficiency, superior quality, and low energy consumption. Micro milling and conventional macro milling share similarities in kinematics, but micro milling differs significantly in several other aspects, including relatively weak stiffness, high spindle speed, radial error motions, and size effects [1,2,3,4,5], which are also the main causes of chatter in micro milling. Chatter is a self-induced vibration between the tool and workpiece that can reduce surface accuracy of the workpiece, generate noise pollution, and significantly shorten the lifespan of both tools and machines [6].

To avoid chatter in the machining process, early research focused on selecting appropriate cutting condition parameters by calculating the stability lobe diagram (SLD). Cutting force is an essential part of solving the SLD. The factors affecting cutting force in micro milling are more complicated. The chip formation is affected by the difference between the thickness of the uncut chip and the radius of the cutting edge due to the size effect; therefore, some characteristics, such as the minimum cutting thickness, chip separation state, and tool runout, should be taken into consideration [7]. Wojciechowski et al. [8] found that chip thickness accumulation significantly affects the dynamic stability of the micro milling process and developed a cutting force model that takes into account the machining system’s geometric error, tool deflection, minimum chip thickness, and chip thickness accumulation. A prediction of cutting forces in micro milling was developed by Wang et al. [9], considering the chip thickness accumulation, the effect of runout, and the dynamic deformations. Wojciechowski et al. [10] investigated the cutting forces from different tool axis inclinations along the tool path for micro ball end milling cutters, and reported that the dynamic stability of the micro ball end milling is influenced by the tool’s axis inclination and feed per tooth. Zhang et al. [3] developed an uncut chip thickness model dedicated to micro end milling that considered the calculation of variable entry and exit angles caused by tool runout and tool deflection. These studies show that seeking an accurate solution for the cutting forces in micro milling presents significant complexity and computational challenges. In addition, the accuracy of SLDs may be limited due to the complex environmental disturbances, variations in cutting conditions, and simplification errors in the cutting system model [11,12,13]. Therefore, the timely detection of chatter is crucial for improving the machining efficiency and quality of parts.

So far, various chatter detection methods have been proposed. Clearly, most of them involve the processes of signal processing and feature extraction [14]. Signal processing techniques play a crucial role in extracting significant chatter features from measured cutting signals. The use of advanced signal processing algorithms can mitigate the effects of low data quality due to limited sensor quality or noises [15]. Researchers have employed various methods in different domains, i.e., the time domain (TD) [16,17], the frequency domain (FD) [18,19], and the time-frequency domain (TFD) [20,21,22,23,24], to extract sensitive chatter features. In addition, some methods, such as recurrence quantification analysis [25,26,27] and multiple fractal analysis [28,29,30], have also been used for chatter detection. Among these techniques, FD- and TFD-based methods are more widely employed. Chang et al. [19] applied fast Fourier transform (FFT) to analyze the spectra of cutting force and vibration signals and proposed a novel FD search algorithm to identify frequency features. However, due to the limitations of FFT in tracking frequency fluctuations over time, TFD methods are necessary for analyzing non-stationary and time-varying signals. Wavelet transform (WT), empirical mode decomposition (EMD), and variational mode decomposition (VMD) are the commonly used TFD methods. EMD is an adaptive signal processing method that can theoretically be applied to various types of signal decompositions, particularly for non-linear and non-smooth signals. However, EMD has seen limited applications in the stability analysis of processing signals due to issues such as insufficient mathematical support, poor interpretability, and the phenomenon of modal mixing. VMD is an adaptive, fully non-recursive approach to modal variational analysis and signal processing. It can efficiently decompose a given signal and obtain an optimal solution to the variational problem. VMD is more robust than EMD in signal preprocessing for chatter detection. Unfortunately, the severe shortcoming of the VMD method lies in the optimal selection of the number of modes *K* and the quadratic penalty α [31]. Additionally, these parameters must be reselected for each specific set of cutting conditions, posing significant challenges to its application in chatter detection. As a time-scale analysis method, WT has a strong ability to characterize local features. However, the poor resolution of WT for the high frequency part affects the analysis of the high frequency region of the signal. To address this limitation, wavelet packet decomposition (WPD) was developed based on WT theory. Zhang et al. [21] combined VMD and WPD to process the cutting force signals and proposed an energy entropy chatter detection method. Based on matrix trap filter and fast WPD, Tao et al. [24] proposed a real-time monitoring method for robotic drilling vibration. Zheng et al. [32] compared the wavelet packet, wavelet leaders multifractal, and p-leader multifractal methods through chatter experiments and pointed out that wavelet packet provides faster computational efficiency.

Feature extraction is typically performed after signal processing. In the machining processes, the onset of chatter is accompanied by distinct changes in signals in both the frequency and energy distributions [33]. Therefore, features related to probability and entropy have been used for chatter detection. Cao et al. [34] applied the Hilbert–Huang spectrum, which is a full time–frequency–energy distribution of the signals to detect chatter. Zhang et al. [35] extracted the sub-signal containing chatter information from VMD, and then realized the online chatter detection using the energy ratio difference in the target sub-signal. Chen et al. [36] removed the frequency components of the spectrum related to the spindle speed and then used Rényi entropy which can effectively characterize the randomness of the spectral distribution to detect chatter. Matthew et al. [37] proposed a wavelet–Hilbert technique and reported an accurate detection of chatter in combination with Rényi entropy. The literature [22,38,39] employed VMD and WPD to extract energy entropy and concluded that energy entropy correlates with chatter vibration and can be used to detect chatter vibration. In fact, energy entropy is a specific form of Shannon entropy [40] and has the widest range of applications among the various applications of Shannon entropy [41]. The Shannon entropy has been adopted to identify chatter in the previous works [20,21,23]. Rényi entropy [42] is a generalized form of Shannon entropy, which can be used to measure information richness and complexity characteristics. It is widely used in various feature recognition [36,43,44].

Traditional methods generally achieve chatter detection by setting a threshold for features, but the threshold relies on a priori knowledge and may not be applicable to other processing systems [45]. Chatter detection can be considered a classification problem and machine learning algorithms can be competent in solving it. Li et al. [31] utilized the angular synchronous averaging technique to obtain the chatter components, then extracted the multi-scale energy entropy and constructed a chatter detection model using gradient tree boosting. Wang et al. [46] decomposed the vibration signals using VMD and extracted the information entropy, then trained an information entropy-based support vector machine model to realize chatter detection. Tran et al. [47] used WPD to decompose both the sound and the vibration signal, performed feature selection based on recursive feature elimination, and built a chatter detection model using an artificial neural network. Sun et al. [48] used successive variational mode decomposition to obtain the energy ratio characteristics of the cutting force signal and constructed a deep attention information fusion network to detect chatter.

According to a recent review [49], there are only a small number of publications focusing on chatter detection and suppression technologies (among approximately 100 pieces of the relevant literature indexed in the Web of Science since 2009). It is worth pointing out that most of the existing chatter detection methods mentioned above mainly target conventional milling processes. The reliability of in-process chatter detection technology is highly dependent on the selected indicators [39]. To achieve more accurate classification of chatter states, choosing appropriate chatter features is indispensable. Thus, determination of highly effective chatter features remains a pressing challenge that requires further investigation.

**Figure 1:**
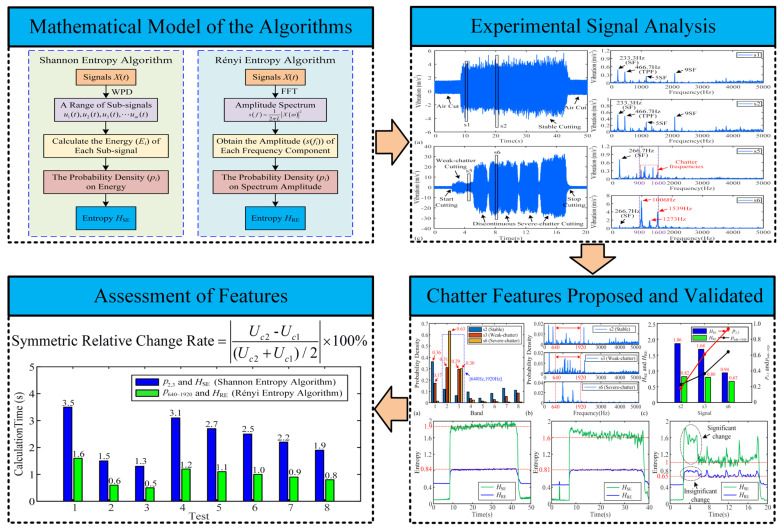
The structural framework of the paper.

In order to identify more sensitive and accurate indicators for chatter detection in micro milling processes, probability- and entropy-related features based on the Shannon and Rényi entropy algorithms are investigated in this study. These features are systematically evaluated based on their change rates, threshold intervals, and computational efficiency. This work aims to provide valuable insights for feature selection in threshold-based and machine learning-based chatter detection methods for micro milling operations. The rest of the paper is organized as follows: Section 2 presents the methodologies of feature extraction employing Shannon entropy and Rényi entropy algorithms. The feasibility and assessment of features for chatter detection are given in Section 3. Section 4 outlines some conclusions drawn from this study. Figure 1 presents the structural framework of this paper.

## 2. Feature Extraction Using Shannon Entropy and Rényi Entropy

Entropy is a quantitative measure of data randomness in a system. Higher entropy values indicate a more uniform and uncertain data distribution, whereas lower entropy values suggest less uncertainty in the distribution. Probability characterizes the likelihood of an event occurring, with higher probability indicating increased certainty of event occurrence. Changes in machining states are reflected in the energy and frequency characteristics of the system. Under stable machining conditions, the energy and frequency distributions remain relatively uniform around the spindle frequency (SF) and its harmonic components. However, during the occurrence of chatter, these distributions are significantly skewed towards the natural frequency of the tool-workpiece system, transforming from a uniform to a concentrated pattern. Given these remarkable characteristics, probability- and entropy-related features provide a promising approach for enhanced machining system monitoring.

### 2.1. Mathematical Model of Shannon Entropy

If a random variable *Y* has *m* possible values, where the *i*th outcome has a probability of *p_i_*, then the Shannon entropy *H*(*p*) is defined as follows [50]:

(1) $$H\left(p\right)=-\sum_{i=1}^{m}p_{i}\log p_{i}$$

**Figure 2:**
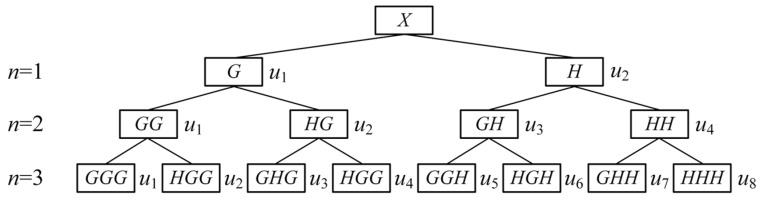
Results of three-layer WPD.

The Shannon entropy algorithm applied in this paper for machining signals relies on WPD. WPD can decompose both the low-frequency parts and the high-frequency parts of a signal *X*. It provides a more refined approach to signal processing, ensuring that the decomposition is neither redundant nor sparse, which enhances time-frequency localization analysis. The structure of a three-layer WPD is illustrated in Figure 2.

**Figure 3:**
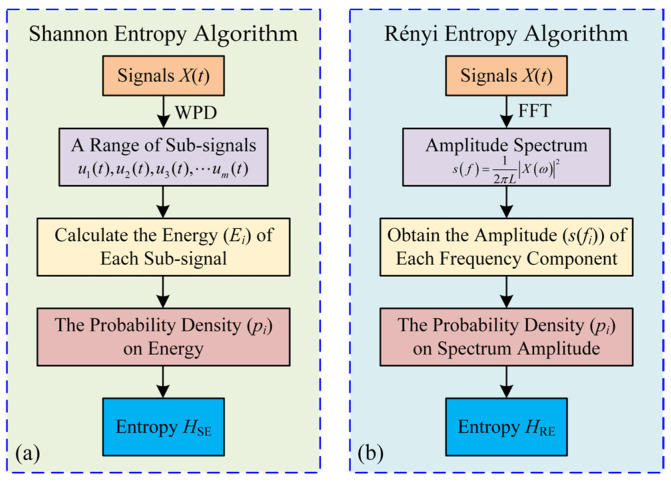
Flow chart of extracting features related to probability and entropy by using (**a**) Shannon entropy, and (**b**) Rényi entropy.

For the time series of machining signal *X*(*t*), *H*_SE_ can be calculated as shown in Figure 3a. Due to two types of entropy being calculated in this paper, the *H*_SE_ and *H*_RE_ are used to denote the result calculated by the Shannon entropy and Rényi entropy algorithms, respectively.

Firstly, perform *n*-layer WPD for the *X*(*t*) to obtain a series of sub-signals *u*_1_(*t*), *u*_2_(*t*), …, *u_m_*(*t*), where *m* = 2*^n^*.

Secondly, the energy *E_i_* of each signal component *u_i_* can be calculated by Equation (2), and the total energy *E* can be obtained by Equation (3).

(2) $$E_{i}=\int_{t1}^{t2}{\left|u_{i}(t)\right|}^{2}dti=1,2,3\ldots m$$

(3) $$E=\sum_{i}^{m}E_{i}$$

where *t*∈[*t*_1_, *t*_2_], *t*_1_, and *t*_2_ are the starting and ending moments of the *X*(t).

Finally, according to Equation (1), the *p_i_* can be generalized to the energy probability density *p_i_* calculated using Equation (4), and the *H*_SE_ of the signal *X*(*t*) can be expressed using Equation (5).

(4) $$p_{i}=\frac{E_{i}}{E}$$

(5) $$H_{\mathrm{SE}}=-\sum_{i=1}^{m}p_{i}\log(p_{i})$$

### 2.2. Mathematical Model of Rényi Entropy

The generalized entropy $H_{\alpha }(p)$ with *α*-order of the *Y* is defined as follows [51]:

(6) $$H_{\alpha }(p)=\frac{1}{1-\alpha }\log_{2}\frac{\sum_{i}p_{i}^{\alpha }}{\sum_{i}p_{i}}\alpha >0,\;\alpha \neq 1$$

For signal *X*(*t*), *H*_RE_ can be calculated as shown in Figure 3b:

Firstly, the spectrum amplitude of *X*(*t*) can be expressed by Equation (7) after FFT:

(7) $$s\left(f\right)=\frac{1}{2\pi L}{\left|X\left(\omega \right)\right|}^{2}$$

where *L* is the length of *X*(*t*) and *X*(*ω*) is the Fourier transform of *X*(*t*).

Secondly, the *p_i_* in Equation (1) can be generalized to the probability density of the spectrum amplitude *p_i_* calculated using Equation (8):

(8) $$p_{i}=\frac{s\left(f_{i}\right)}{\sum_{i=1}^{M}s\left(f_{i}\right)}i=1,2,3,\ldots ,M$$

where *s*(*f_i_*) is the amplitude of the frequency component *f_i_* and *p_i_* is the corresponding probability density. *M* is the number of frequency components of the spectrum.

Finally, according to Equation (6), the *H*_RE_ of the signal *X*(*t*) can be expressed as follows:

(9) $$H_{\mathrm{RE}}=-\frac{\log_{2}\sum_{1}^{M}{\left(p_{i}\right)}^{3}}{2\cdot \log_{2}M}$$

where *α* = 3 was chosen according to the literature [36].

## 3. Experimental Assessment and Discussion

### 3.1. Experimental Setup

**Figure 4:**
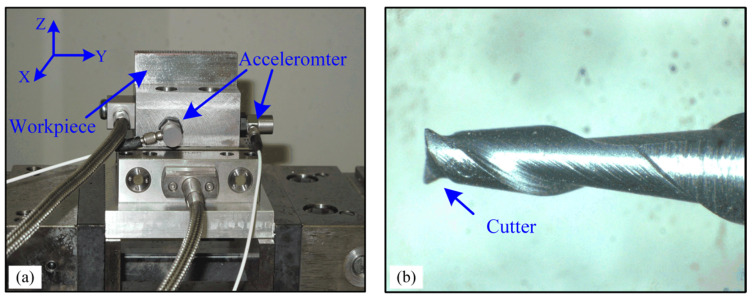
Experimental setup: (**a**) workpiece and accelerometer mounted on the CNC work table, (**b**) micro end mill cutter.

As shown in Figure 4a, the micro milling experiments were carried out on a 5-axis CNC machining center (DECKEL MAHO DMU 60P, DMG MORI, Bielefeld, Germany). The workpiece is made of AISI 1040 carbon steel and designed with a base and a rectangular block of 60 mm × 8 mm × 18 mm on the top. The X- and Y-axis refer to the feed direction and the cross-feed direction, respectively. *Z*-axis is the rotational axis of the cutter. Two ICP accelerometers (PCB Model 352C33, PCB Piezotronics, Depew, NY, USA) with a sensitivity of 100 mV/g were installed on the side of the workpiece in X- and Y- directions to collect vibration signals at a sampling frequency (*fs*) of 10 k Hz. A 600 μm diameter two-flute flat end mill tool was used in the milling process, and the rake and helix angle are 10° and 30°, respectively, as shown in Figure 4b.

The material removal mechanics in micro milling are influenced by the relationship between the instantaneous uncut chip thickness and the minimum chip thickness. Kim [52] proposed that the minimum chip thickness is 30% of the cutting-edge radius. The cutting-edge radius of the selected tool in this study is about 3.8 μm; therefore, the minimum chip thickness can be approximated as 1.2 μm. The three major condition variables in micro milling, i.e., spindle speed, feed rate, and depth of cut, are the main variables affecting the material removal process [53]. In this study, the micro milling experiments were designed with the same depth of cut but different feeds per tooth and spindle speeds. The experiment parameters are listed in Table 1. As it can be seen, the values of the feed per tooth include values both below and above the minimum chip thickness. To make sure each test has the same depth of cut, it is necessary to cut a zero-cutting plane before machining real surfaces. A clean-cut was performed on the workpiece using an 8 mm tool before the experiment. Table 2 lists the natural frequencies of the tool-spindle obtained by impact testing in Ref. [29].

### 3.2. TD and FD Analysis of Experimental Tests

**Figure 5:**
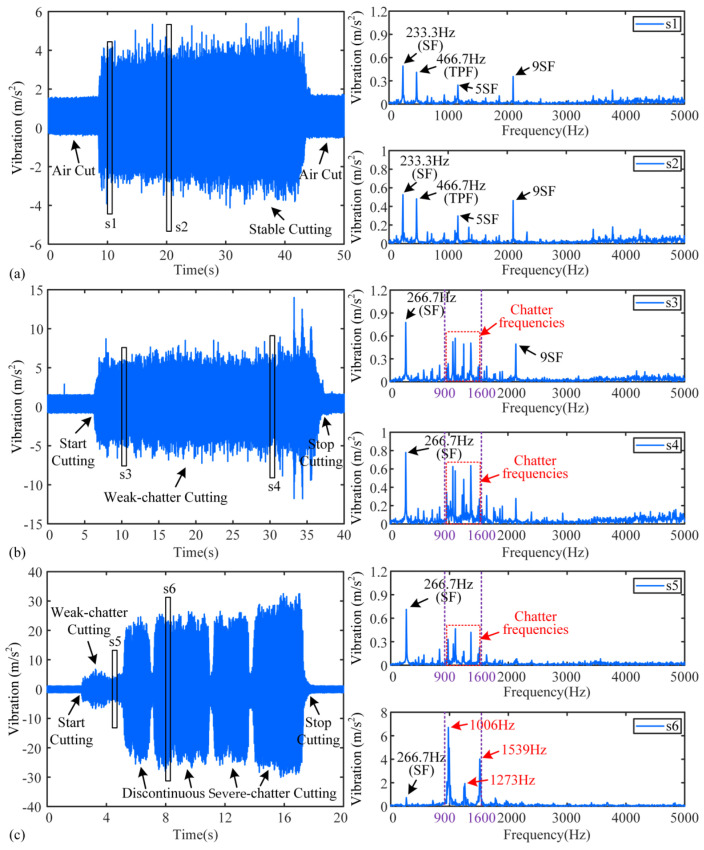
TD and FD diagrams of the three tests: (**a**) test 1, (**b**) test 4, (**c**) test 8.

The X-direction vibration signals of tests are analyzed, and Figure 5 shows the TD and FD of Test 1,4, and 8, where s1–s6 are signal fragments taken from the three tests. In test1, the vibration amplitude in TD of s1 and s2 is less than 5 m/s^2^, and the SF, tool passing frequency (TPF), and their harmonics in the spectrum are the dominant frequencies. No chatter frequency appeared in the FD of s1 and s2, thus, they are stable cutting stages. Small amplitude stray frequencies that appear in the spectrum may be caused by noise and slight vibration. In test 4, the vibration amplitude increases noticeably compared to that of test 1, and it is larger than 5 m/s^2^ and smaller than 10 m/s^2^, except in 33–36 s. The FDs of s3 and s4 in test 4 are shown in Figure 5b, and although SF is still the dominant peak, the chatter frequencies appear. Due to the small amplitude of the chatter frequencies in the spectrum, these stages can be considered weak-chatter. The cutting process of test 8 consisted of two phases: the first cutting phase started at 2.5 s and lasted until 5.5 s, with the vibration amplitude fluctuating at 7.5 m/s^2^; the second phase lasted from 5.5 s to the end of milling and the vibration amplitude increased to 20~30 m/s^2^. The signal fragments s5 and s6 are selected from the two phases, respectively, and their FDs are given as shown in Figure 5c. The FD of s5 is similar to that of s3 and s4; thus, it is a weak-chatter stage. However, the FD of s6 exhibits significant variations. Frequencies of 1006 Hz, 1273 Hz, and 1539 Hz become the dominant peaks in the FD, and the difference in the dominant peaks is close to 266.7 Hz, which suggests that spindle modulation occurs during machining. At this stage, the amplitude of the chatter frequencies is significantly greater than the stable frequency and thus considered severe-chatter.

The occurrence of chatter in micro milling is affected not only by the depth of cut and spindle speed but also by the size effects [49]. The cutting conditions of the eight tests, including the chatter stability and the signal features, are presented in Appendix A. The stability of the cutting process demonstrates a strong correlation between the feed per tooth and the minimum chip thickness. When the feed per tooth is greater than the minimum chip thickness (tests 2 and 3), the milling process shows good stability due to the continuous cutting process and stable material removal. However, when the feed per tooth was close to the minimum chip thickness (tests 5 to 8), chatter occurred in the cutting process. This instability mainly originated from the critical state of machining, where cutting and plowing co-existed, leading to fluctuations in the process damping characteristics that induce chatter. For the cases where the feed per tooth is significantly below the minimum chip thickness (tests 1 and 4), the machining stability exhibits sensitivity to the spindle speed. This is due to the fact that when the spindle speed is relatively low, process damping coefficients gradually increase as the tool wears, consequently enhancing the process damping force and machining stability. However, this stabilizing effect diminishes as the spindle speed increases [49].

### 3.3. Assessment of Proposed Features from Shannon Entropy and Rényi Entropy

**Figure 6:**
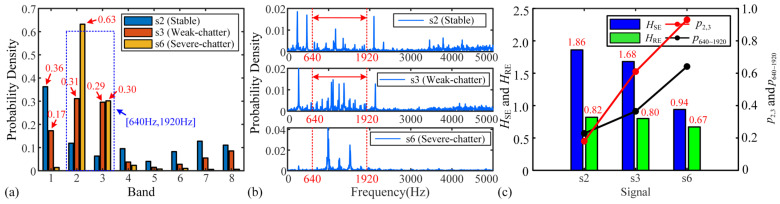
Probability density and entropy for s2, s3, and s6: (**a**) probability density of the energy of the Shannon entropy algorithm, (**b**) probability density of the spectrum amplitude of he Rényi entropy algorithm, and (**c**) probability- and entropy-related features.

The probability density of the energy and spectrum amplitudes of s2, s3, and s6 are shown in Figure 6a,b. In the Shannon entropy algorithm, the energy probability density of each band is determined by the frequency components within its respective interval. Each frequency interval can be calculated according to $\left[(i-1)\cdot \frac{fs}{2\cdot 2^{n}},i\cdot \frac{fs}{2\cdot 2^{n}}\right]$, where *i* and *n* denote the bands and the number of decomposition layers, respectively. In this paper, Daubechies wavelet (db10) is selected to use in WPD, and the signals are decomposed into three layers [21]. Db10 has highly vanishing moments, good smoothing, and balanced time-frequency resolution. There will be 8 sub-signals, or 8 bands, produced by the original signal after decomposition. In the Rényi entropy algorithm, the probability density of each frequency component is dependent on its amplitude in the FD. According to Figure 5, the chatter frequencies mainly appear in the range of 900 Hz to 1600 Hz, so the bands in which the chatter frequencies are located are band 2 [640 Hz, 1280 Hz] and band 3 [1280 Hz, 1920 Hz] in the Shannon entropy algorithm. However, the frequency interval [640 Hz, 1920 Hz] is enlarged relative to [900 Hz, 1600 Hz]. Therefore, to ensure the reasonableness of the assessment, for the Rényi entropy algorithm, the frequency interval [640 Hz, 1920 Hz] is also selected as the frequency interval of the chatter frequencies.

According to Figure 6a, for s2, the probability density distribution remains relatively uniform across all frequency bands except band 1. This uniformity results in a maximum *H_SE_*, as shown in Figure 6c. The elevated probability density in band 1 is attributed to the presence of the dominant frequencies SF and TPF, which making more energy present in this band relative to the other bands. For s3, the presence of chatter frequencies triggers the transfer of energy to the corresponding frequency bands, resulting in an increase in the probability density to 0.31 and 0.29 for bands 2 and 3, respectively. At this time, the probability density is mainly distributed in bands 1 to 3, suggesting that a more localized distribution pattern is beginning to emerge. This makes it a lower *H*_SE_ compared to s2. While for s6, the chatter frequencies become dominant, at this point, the energy of the system is completely transferred to the bands in which the chatter frequencies are located. Consequently, the probability density becomes highly concentrated in bands 2 and 3 with values of 0.63 and 0.30, which lead to a minimum *H*_SE_. Figure 6c illustrates the results of the sum ($p_{2,3}$) of probability densities for bands 2 and 3 in s2, s3, s6. It can be observed that $p_{2,3}$ and *H*_SE_ change with the change in cutting states, so they can be considered features for chatter detection.

The probability density of the spectral amplitude is shown in Figure 6b. Similarly to Figure 6a, the probability density of the frequency components in the interval [640 Hz, 1920 Hz] gradually increases with the change in cutting state. When severe-chatter occurs, the probability density exists almost only in the interval [640 Hz, 1920 Hz] in the whole FD, and the probability density distribution appears to be very concentrated. *H*_RE_ and the sum ($p_{640\sim 1920}$) of the probability densities in the frequency intervals [640 Hz, 1920 Hz] are calculated for s2, s3, and s6, as shown in Figure 6c. The *H_RE_* for stable, weak- and severe- chatter are 0.82, 0.80, and 0.67, respectively. The variation in the *H*_RE_ for stable and weak-chatter is not significant, suggesting that it may not be available for identifying weak-chatter. On the contrary, $p_{640\sim 1920}$ shows significant changes in the three cutting states, which suggests that it can be used to recognize different cutting states similar to $p_{2,3}$ and *H*_SE_.

To perform real-time analysis of signals, a sliding window of length 2000 was used to process the eight tests signals with a window step of 1000. The four features $p_{2,3}$, $p_{640\sim 1920}$, *H*_SE_, and *H*_RE_ were extracted under each window and the results can be viewed in Appendix A.

**Figure 7:**
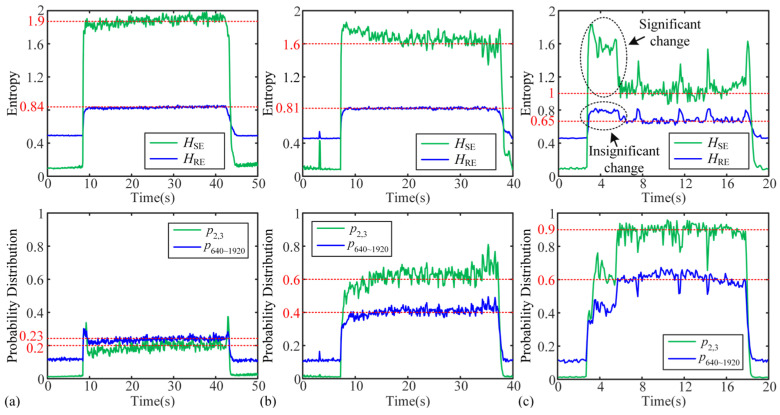
Real-time analysis for tests 1, 4, 8: (**a**) test 1, (**b**) test 4, and (**c**) test 8.

Figure 7 shows the results of the real-time analysis for tests 1, 4, and 8. Since test 1 is a stable cutting process, the fluctuations of the four features throughout the time period are small, distributed at 1.9, 0.84, 0.2, and 0.23, respectively. For test 4, all three features except *H*_RE_ show some fluctuations, especially at 33–36 s, which is consistent with the analysis results in the TD. Among them, the variation of *H*_SE_ and $p_{2,3}$ is significantly larger than *H*_RE_ and $p_{640\sim 1920}$, which indicates that *H*_SE_ and $p_{2,3}$ have better sensitivity. In addition, due to the fact that this cutting process is a weak-chatter cutting process, the real-time results of the four features change relative to test 1, which are distributed at 1.6, 0.81, 0.6, 0.4, respectively. In Figure 7c, since test 8 contains both weak-chatter and discontinuous severe-chatter cutting processes, the amplitudes of the four features appear to change significantly at the moment of cutting state changes, especially *H*_SE_ and $p_{2,3}$. During the discontinuous severe-chatter process, the amplitudes of the four features fluctuate at 1.0, 0.65, 0.9, and 0.6, respectively.

The above analysis shows that all the four proposed features can be used for real-time chatter detection.

To select the most suitable chatter features, the proposed features were systematically assessed from feature sensitivity, threshold interval, and computation time. The sensitivity of the four features to different levels of chatter can calculated according to Equation (10).

(10) $$\mathrm{Symmetric}\;\mathrm{Relative}\;\mathrm{Change}\;\mathrm{Rate}=\left|\frac{U_{c2}-U_{c1}}{(U_{c2}+U_{c1})/2}\right|\times 100\%$$

where *c*1 and *c*2 are used to represent the two states before and after the change in machining state, and *U* is used to denote the features.

Table 3 lists the values of $p_{2,3}$, $p_{640\sim 1920}$, *H*_SE_, and *H*_RE_ and their change rates of the three tests according to Figure 7. According to Appendix A, the *H*_RE_ calculated from both the stable and weak-chatter tests is around 0.8, which is not applicable for the identification of weak-chatter. Therefore, there is no discussion of this metric in Table 3 for the identification of weak-chatter. For the probability-related features, $p_{2,3}$ demonstrates excellent feature recognition ability with change rates up to 100%, 40%, and 127.27%, especially from stable- to weak-chatter and stable- to severe-chatter. The change rates of $p_{640\sim 1920}$ are 53.97%, 40%, and 89.16%, respectively, which is significantly smaller than $p_{2,3}$. This suggests that $p_{2,3}$ is more sensitive to chatter than $p_{640\sim 1920}$. For the entropy related features, the change rates of *H*_SE_ across the three states are 17.14%, 46.15%, and 62.07%, while *H*_RE_’s change rate is 25.5%. This shows that *H*_SE_ is sensitive to changes in weak- to severe-chatter and stable- to severe-chatter while insensitive to the change in stable- to weak-chatter; *H*_RE_ is only sensitive to changes in stable- to severe-chatter. Therefore, by comparing the change rates of the features, it can be concluded that the features extracted from Shannon entropy algorithm are more sensitive to different levels of chatter than those extracted from Rényi entropy algorithm. For probability-related and entropy-related features extracted from the same algorithm, the probability related features demonstrate greater change rates, whether it is the Shannon entropy algorithm or the Rényi entropy algorithm, which indicates that it is more sensitive to chatter.

The features from the Shannon entropy algorithm characterize signal state variations through calculating the energy distribution of the signal in different frequency bands. Due to the significant fluctuations of the signal in the time domain caused by chatter during the micro milling process, the features calculated by the Shannon entropy algorithm have a high sensitivity. In comparison, the Rényi entropy algorithm generates features through frequency domain information. For weak-chatter signals with subtle spectral variations, the calculated features exhibit minimal changes. Conversely, characterized by pronounced spectral alterations, severe-chatter conditions result in more distinct feature variations using the Rényi entropy algorithm.

According to the eight test results in Appendix A, Table 4 provides the threshold for the features of different cutting states. The threshold ranges of $p_{2,3}$ and $p_{640\sim 1920}$ can well distinguish stable-, weak-chatter, and severe-chatter states. For *H*_SE_, there exists confusion between stable- and weak-chatter in some cases, such as the 7–13 s of test 4. The threshold of *H*_RE_ can only be used to distinguish stable- and severe-chatter.

**Figure 8:**
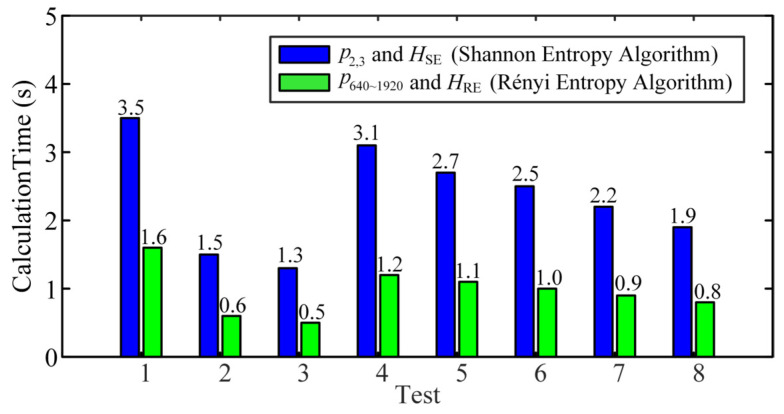
Calculation time of the four features for multi-group tests.

A 10-core 64-bit computer was selected in conjunction with MATLAB software to perform the Shannon entropy and Rényi entropy algorithms in real-time, and the calculation time required for the eight tests is shown in Figure 8. For the same test, Rényi entropy algorithm performed its calculations in a shorter timeframe and more efficiently. For different tests, the length of the signal data influenced the computation time, while the Shannon entropy algorithm was more affected. In addition, it can be noticed that the times of the real-time computation of each test are much shorter than the length of the signal’s own time, which indicates that they have a good potential for practical engineering applications. Due to the small difference in computation time of features under the same algorithm, the computation times of $p_{2,3}$ and *H*_SE_ and $p_{640\sim 1920}$ and *H*_RE_ were not given separately.

## 4. Conclusions

In this study, four chatter features extracted from the Shannon entropy and Rényi entropy algorithms for micro milling process were presented and assessed. Their feasibility for chatter detection was validated using actual machining tests under stable-, weak-chatter, and severe-chatter conditions. The four features are assessed based on their change rates, threshold intervals, and calculation times. Some conclusions that can be drawn from the results of this study are as follows:

- Four features that can be used for real-time chatter detection are extracted from the Shannon entropy and Rényi entropy algorithms and their thresholds are given separately.
- The probability-related feature ($p_{2,3}$) extracted from the Shannon entropy algorithm exhibits the highest chatter sensitivity in a variety of situations.
- The results show that probability-related features are more sensitive and have the potential to be applied in chatter detection rather than entropy-related features for the same algorithm.
- The features extracted from Shannon entropy algorithm are more sensitive to chatter, while the features extracted from the Rényi entropy algorithm show some applicability to chatter detection with minimum computation time.

Based on the feature extraction method developed in this study, it is expected that an adaptive control system can be developed and integrated into the CNC systems in the future. In such a system, process stability monitoring and chatter onset detection can be implemented by employing the probability-related features from the Shannon entropy algorithm, which are highly sensitive to chatter, while utilizing the more computationally efficient features of the Rényi entropy algorithm as supplementary indicators. When a change in chatter feature is detected, the system will promptly adjust critical machining parameters, such as cutting depth and spindle speed, to suppress chatter. Therefore, this study is expected to contribute to the optimization of the manufacturing process, which in turn improves the overall performance and industry competitiveness.

## Figures and Tables

**Table 1:** Experiment parameters.

Test	Depth of Cut (μm)	Feed Per Tooth (μm/Tooth)	Spindle Speed (rpm)
1	50	0.5	14,000
2	50	2.0	14,000
3	50	2.0	16,000
4	50	0.5	16,000
5	50	1.0	10,000
6	50	1.0	12,000
7	50	1.0	14,000
8	50	1.0	16,000

**Table 2:** Natural frequencies of the tool-spindle.

	Direction	Natural Frequency (Hz)
Tool-spindle	X, Y	1344, 1920, 2896, 4768

**Table 3:** The $p_{2,3}$, $p_{640\sim 1920}$, *H*_SE_, and *H*_RE_ and their change rates in tests 1, 4, and 8.

SE and RE		$p_{2,3}$	$p_{640\sim 1920}$	*H*_SE_	*H*_RE_
Cutting States and Change Rates					
Cutting states	Stable	0.2	0.23	1.9	0.84
	Weak-chatter	0.6	0.4	1.6	-
	Severe-chatter	0.9	0.6	1.0	0.65
Change rates for probability distribution and entropy (%)	Stable to weak-chatter	100	53.97	17.14	-
	Weak-chatter to severe-chatter	40	40	46.15	-
	Stable to severe-chatter	127.27	89.16	62.07	25.5

**Table 4:** Threshold for the features to different cutting states.

	$p_{2,3}$	$p_{640\sim 1920}$	*H*_SE_	*H*_RE_
Stable	<0.4	<0.3	>1.7	>0.75
Weak-chatter	0.4~0.8	0.3~0.45	1.4~1.7	-
Severe-chatter	>0.8	>0.45	<1.4	<0.75

## Data Availability

Data are contained within the article.

## Nomenclature

*Acronyms*	
EMD	empirical mode decomposition
FD	frequency domain
FFT	fast Fourier transform
RE	Rényi entropy
SE	Shannon entropy
SF	spindle frequency
SLD	stability lobe diagram
TD	time domain
TFD	time-frequency domain
TPF	tool passing frequency
VMD	variational mode decomposition
WPD	wavelet packet decomposition
WT	wavelet transform
*Symbols*	
*c*1, *c*2	the two states before and after the change in machining state
*E*	energy of signal
*f*	frequency component
*fs*	sampling frequency
*H*_SE_	Shannon entropy
*H*_RE_	Rényi entropy
*L*	length of *X*(*t*)
*n*	layer of WPD
*M*	the number of frequency component of the spectrum
*p*	probability
*p*_2,3_	the sum of probability densities for bands 2 and 3
*p*_640~1920_	the sum of the probability densities in the frequency intervals [640 Hz, 1920 Hz]
*s*(*f*)	amplitude of the frequency component *f*
*u*_i_(*t*)	sub-signal of X after WPD
*U*	features
*X*, *X*(*t*), s1–s6	signal
*X*(*ω*)	Fourier transform of *X*(*t*)
*Y*	random variable

## Appendix A

**Test and Containing State**	**Time Domain of Signals**	**Features of Shannon Entropy Algorithm**	**Features of Rényi Entropy Algorithm**
1 Stable			
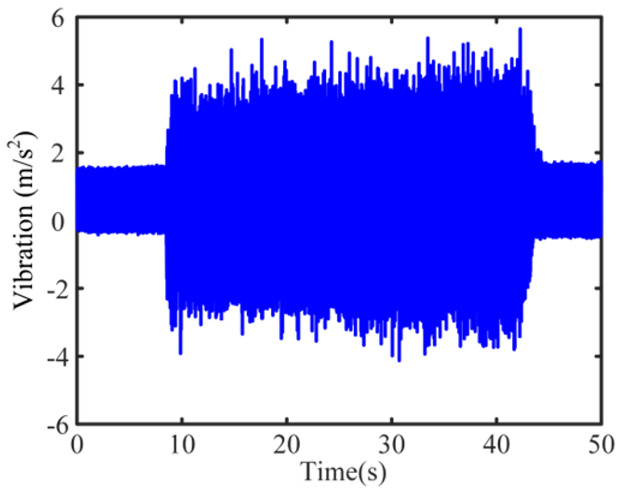
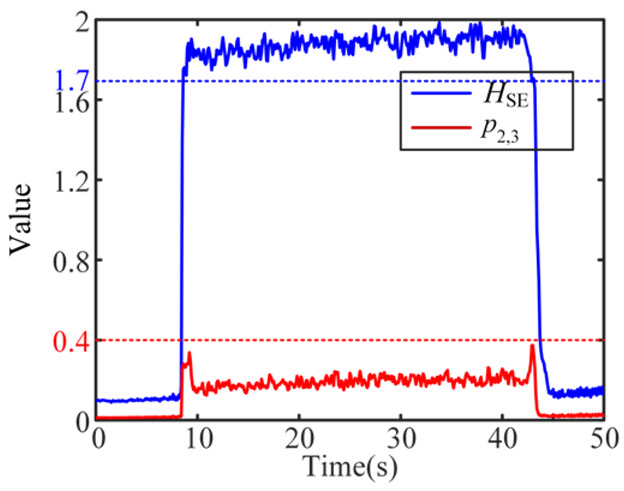
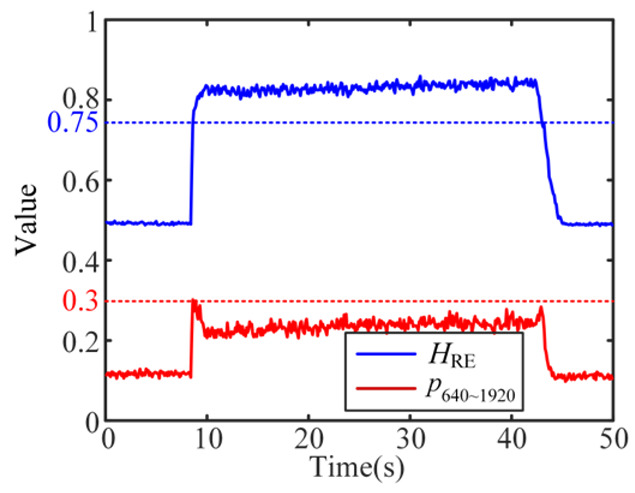
2 Stable			
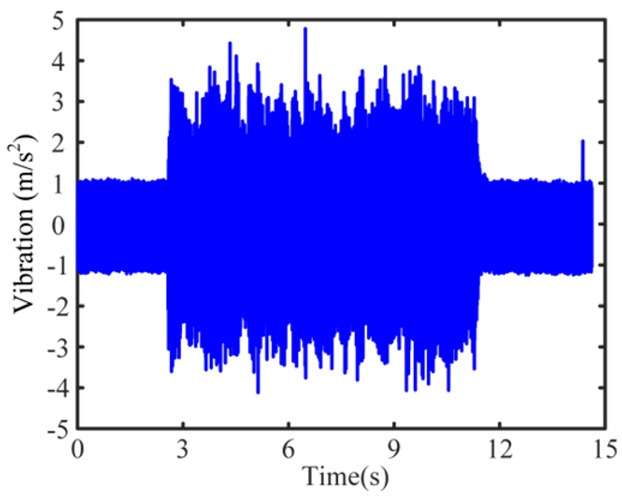
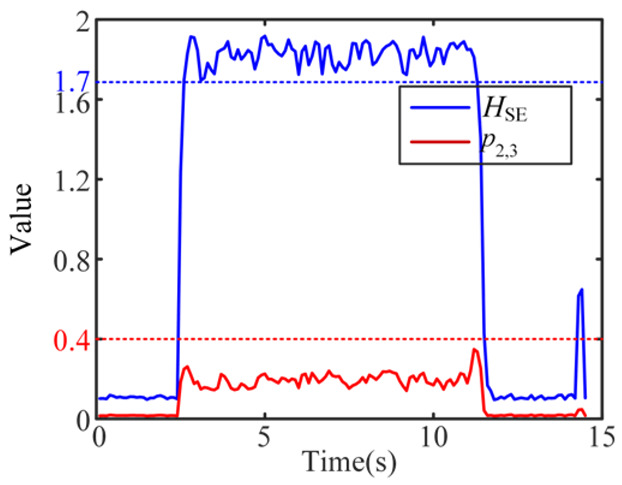
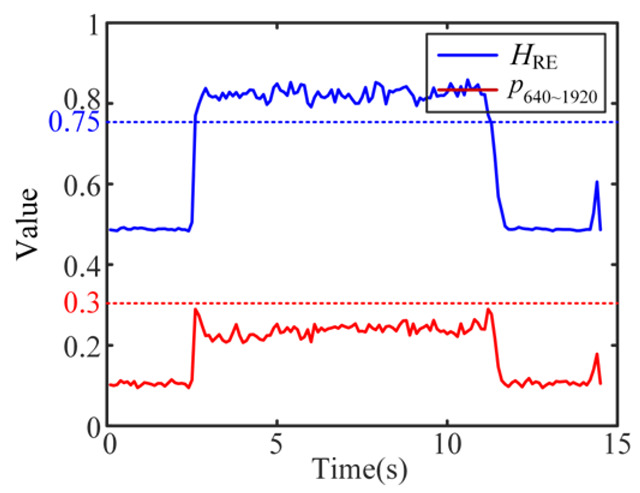
3 Stable			
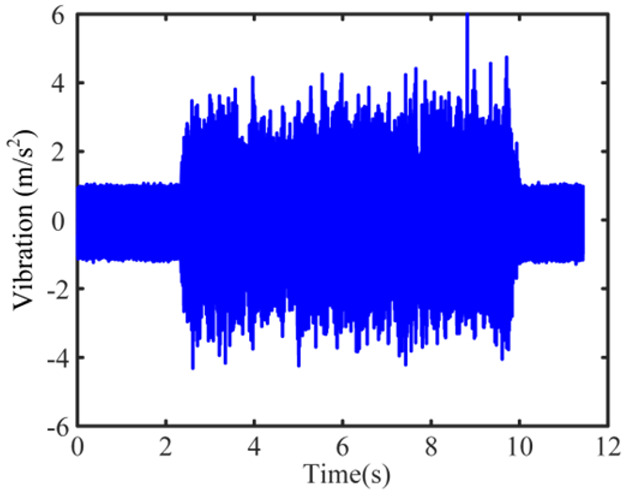
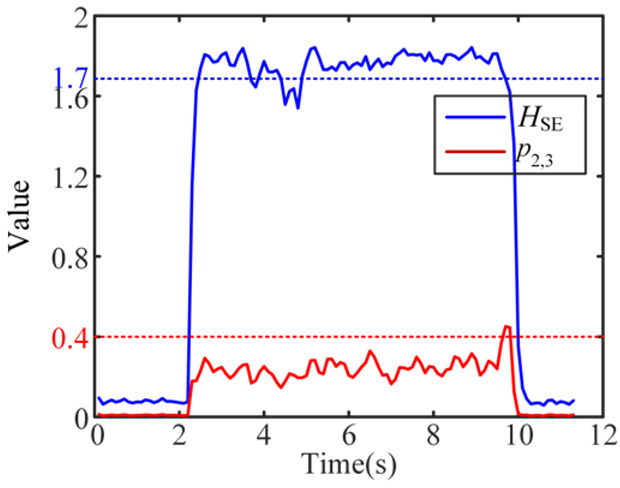
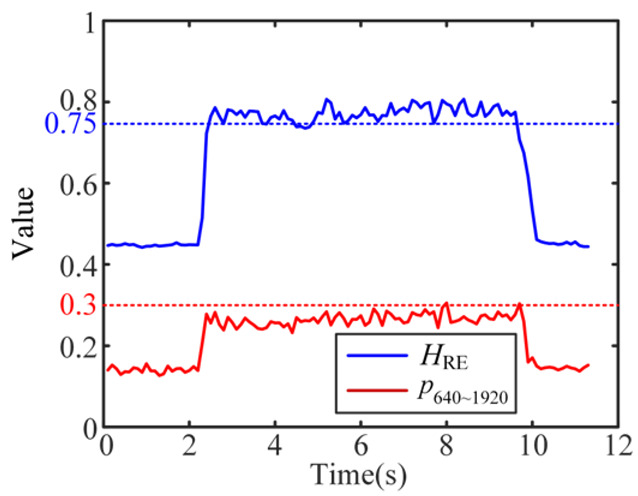
4 Weak-chatter			
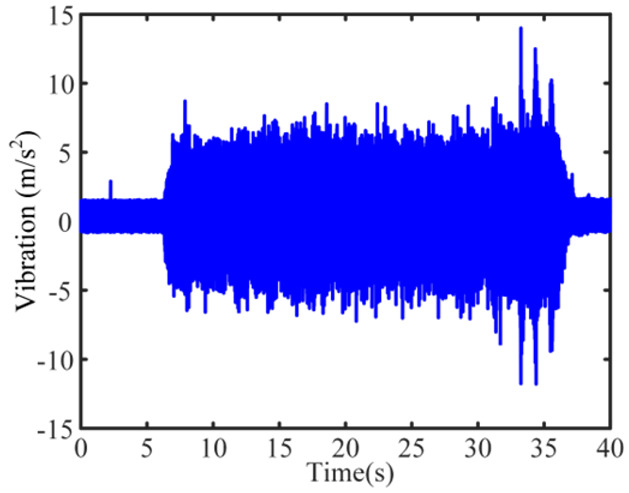
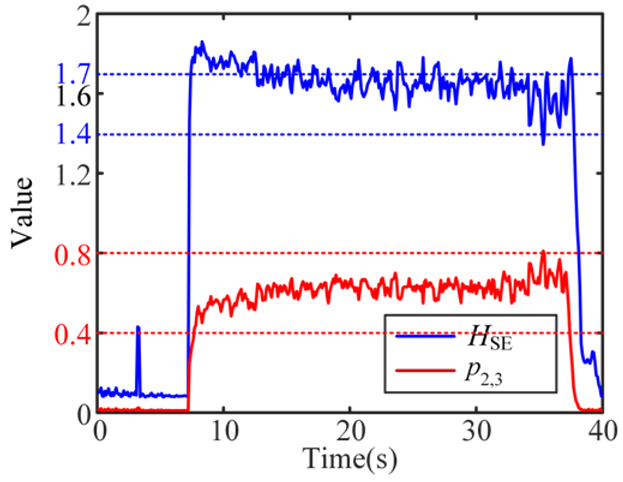
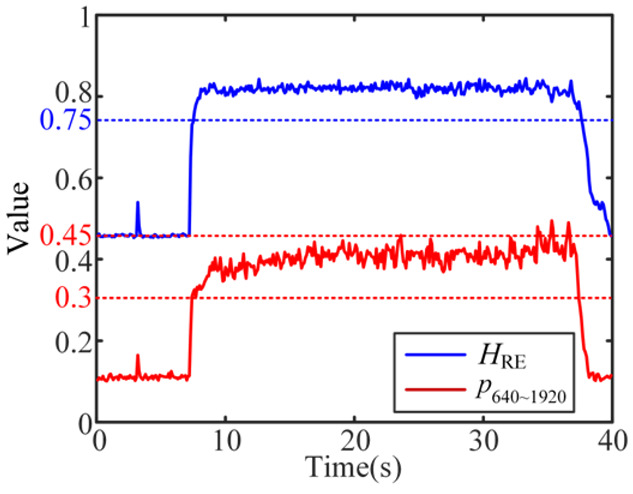
5 Severe-chatter			
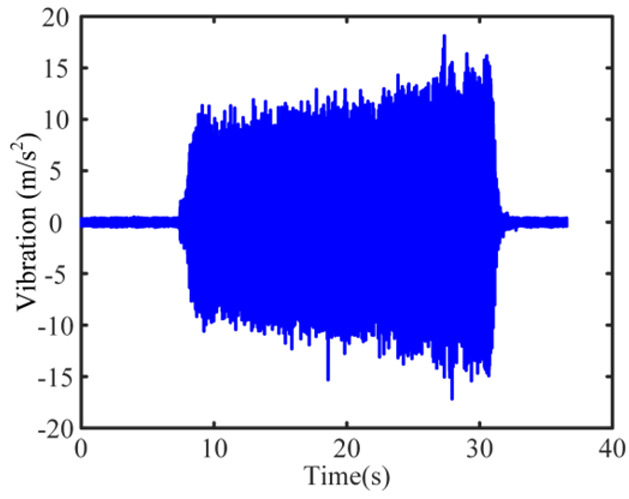
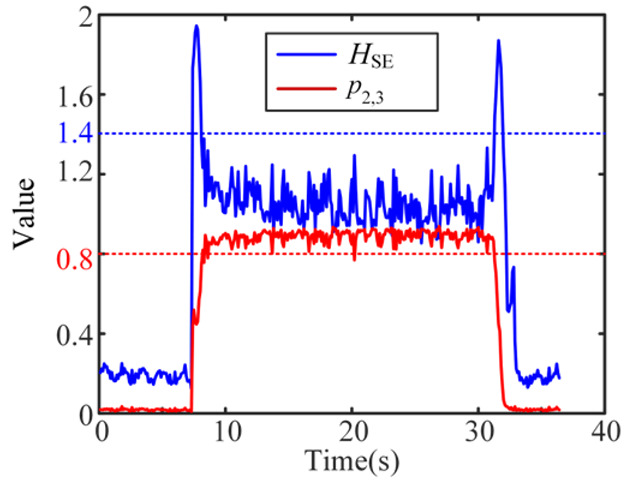
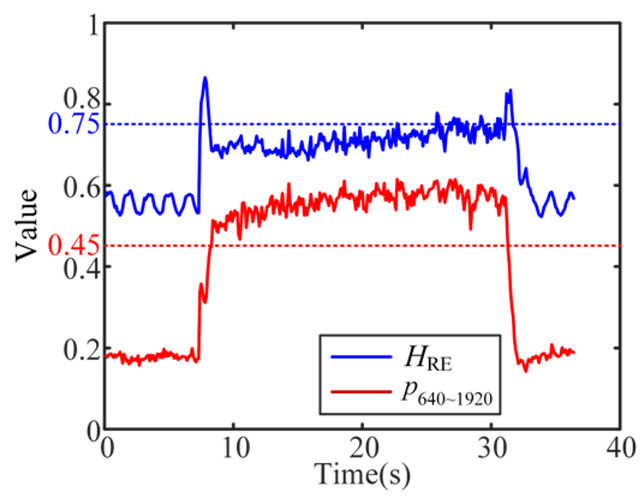
6 Severe-chatter			
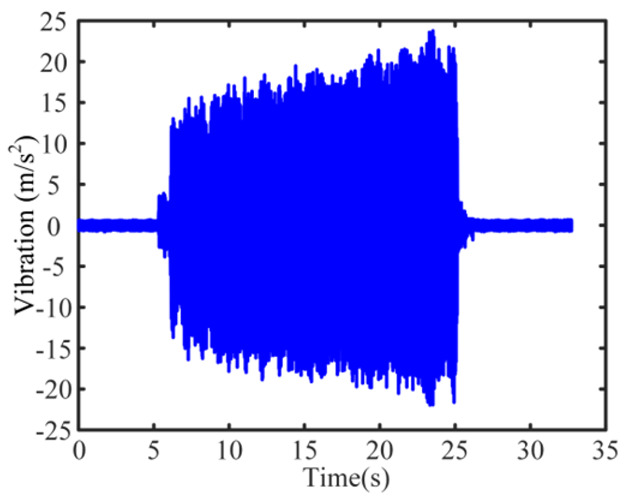
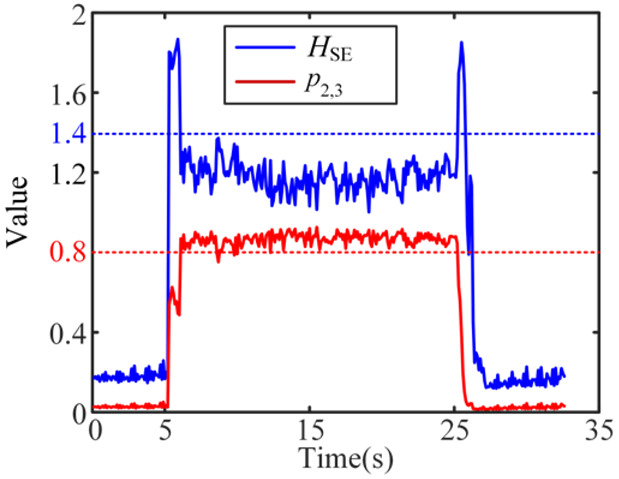
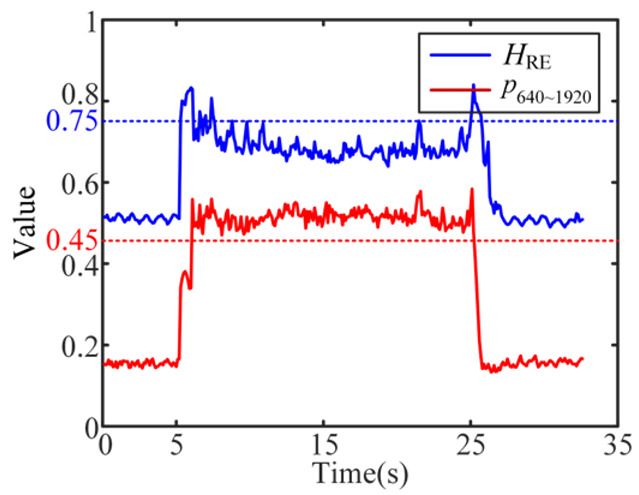
7 Stable and Severe-chatter			
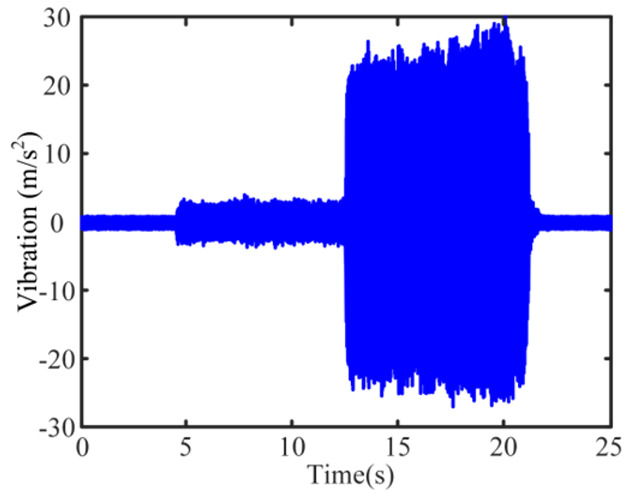
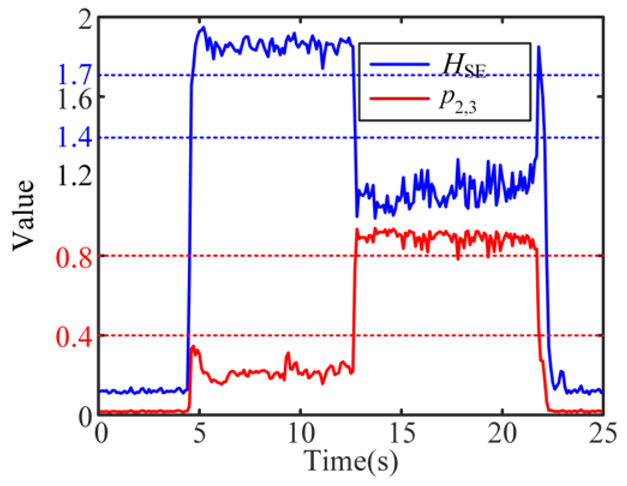
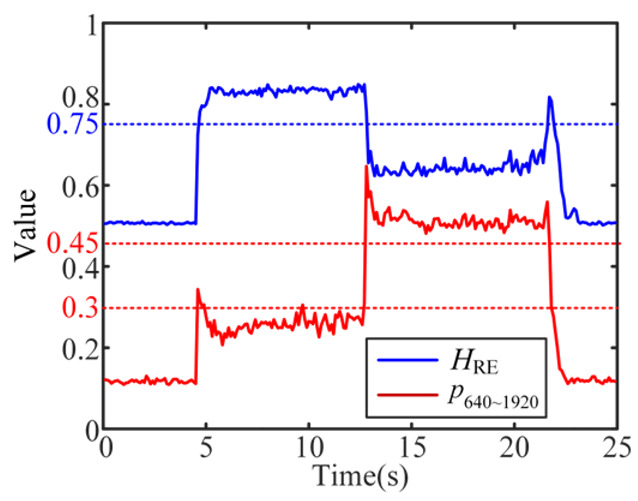
8 Weak-chatter and Severe-chatter			

## References

[1] Chen N., Li H.N., Wu J., Li Z. (2021). Advances in micro milling: From tool fabrication to process outcomes. Int. J. Mach. Tools Manuf..

[2] Balazs B.Z., Geier N., Takacs M., Davim J.P. (2021). A review on micro-milling: Recent advances and future trends. Int. J. Adv. Manuf. Technol..

[3] Zhang X., Ehmann K.F., Yu T., Wang W. (2016). Cutting forces in micro-end-milling processes. Int. J. Mach. Tools Manuf..

[4] Shekhar S., Bediz B., Ozdoganlar O.B. (2023). Tool-tip dynamics in micromachining with arbitrary tool geometries and the effect of spindle speed. Int. J. Mach. Tools Manuf..

[5] Shekhar S., Nahata S., Ozdoganlar O.B. (2020). The effect of spindle dynamics on tool-tip radial throw in micromachining. J. Manuf. Process..

[6] Özşahin O., Budak E., Özgüven H.N. (2015). In-process tool point FRF identification under operational conditions using inverse stability solution. Int. J. Mach. Tools Manuf..

[7] Ding P., Huang X., Miao X., Li S., Liu H. (2023). Dynamic stability simulation of micro-milling under the condition of multi-parameter uncertainty. Probabilistic Eng. Mech..

[8] Wojciechowski S., Matuszak M., Powałka B., Madajewski M., Maruda R.W., Królczyk G.M. (2019). Prediction of cutting forces during micro end milling considering chip thickness accumulation. Int. J. Mach. Tools Manuf..

[9] Wang P., Bai Q., Cheng K., Zhao L., Ding H. (2022). The Modelling and Analysis of Micro-Milling Forces for Fabricating Thin-Walled Micro-Parts Considering Machining Dynamics. Machines.

[10] Wojciechowski S., Mrozek K. (2017). Mechanical and technological aspects of micro ball end milling with various tool inclinations. Int. J. Mech. Sci..

[11] Yesilli M.C., Khasawneh F.A., Otto A. (2020). On transfer learning for chatter detection in turning using wavelet packet transform and ensemble empirical mode decomposition. CIRP J. Manuf. Sci. Technol..

[12] Cao H., Zhang X., Chen X. (2017). The concept and progress of intelligent spindles: A review. Int. J. Mach. Tools Manuf..

[13] Postel M., Özsahin O., Altintas Y. (2018). High speed tooltip FRF predictions of arbitrary tool-holder combinations based on operational spindle identification. Int. J. Mach. Tools Manuf..

[14] Wang W.-K., Wan M., Zhang W.-H., Yang Y. (2022). Chatter detection methods in the machining processes: A review. J. Manuf. Process..

[15] Stavropoulos P., Souflas T., Manitaras D., Papaioannou C., Bikas H. (2023). Optimization of Milling Processes: Chatter Detection via a Sensor-Integrated Vice. Machines.

[16] Shao Y., Deng X., Yuan Y., Mechefske C.K., Chen Z. (2014). Characteristic recognition of chatter mark vibration in a rolling mill based on the non-dimensional parameters of the vibration signal. J. Mech. Sci. Technol..

[17] Lamraoui M., Barakat M., Thomas M., el Badaoui M.M. (2015). Chatter detection in milling machines by neural network classification and feature selection. J. Vib. Control.

[18] Shao Q., Feng C. (2011). Pattern recognition of chatter gestation based on Hybrid PCA-SVM. Appl. Mech. Mater..

[19] Chang L., Weiwei X., Lei G. (2020). Identification of milling chatter based on a novel frequency-domain search algorithm. Int. J. Adv. Manuf. Technol..

[20] Yang K., Wang G., Dong Y., Zhang Q., Sang L. (2019). Early chatter identification based on an optimized variational mode decomposition. Mech. Syst. Signal Process..

[21] Zhang Z., Li H., Meng G., Tu X., Cheng C. (2016). Chatter detection in milling process based on the energy entropy of VMD and WPD. Int. J. Mach. Tools Manuf..

[22] Liu C., Zhu L., Ni C. (2018). Chatter detection in milling process based on VMD and energy entropy. Mech. Syst. Signal Process..

[23] Ji Y., Wang X., Liu Z., Wang H., Jiao L., Wang D., Leng S. (2018). Early milling chatter identification by improved empirical mode decomposition and multi-indicator synthetic evaluation. J. Sound Vib..

[24] Tao J., Qin C., Xiao D., Shi H., Liu C. (2019). A pre-generated matrix-based method for real-time robotic drilling chatter monitoring. Chin. J. Aeronaut..

[25] Rusinek R., Lajmert P. (2020). Chatter Detection in Milling of Carbon Fiber-Reinforced Composites by Improved Hilbert–Huang Transform and Recurrence Quantification Analysis. Materials.

[26] Rajesh V.G., Narayanan Namboothiri V.N. Utilizing recurrence quantification analysis for chatter detection in turning. Proceedings of the ASME 2010 International Manufacturing Science and Engineering Conference.

[27] Elias J., Narayanan Namboothiri V.N. (2014). Cross-recurrence plot quantification analysis of input and output signals for the detection of chatter in turning. Nonlinear Dyn..

[28] Chen Y., Li H.Z., Hou L., Bu X.J., Ye S.G., Chen D. (2022). Chatter detection for milling using novel p-leader multifractal features. J. Intell. Manuf..

[29] Jing X., Zheng Z., Xu J., Wang F., Jaffery S.H.I., Li H. (2022). Stability analysis in micro milling based on p-leader multifractal method. J. Manuf. Process..

[30] Ji Y., Wang X., Liu Z., Yan Z., Jiao L., Wang D., Wang J. (2017). EEMD-based online milling chatter detection by fractal dimension and power spectral entropy. Int. J. Adv. Manuf. Technol..

[31] Li K., He S., Li B., Liu H., Mao X., Shi C. (2020). A novel online chatter detection method in milling process based on multiscale entropy and gradient tree boosting. Mech. Syst. Signal Process..

[32] Zheng Z., Jing X., Wang Y., Song X., Li H. (2023). A Comparison of Wavelet Packet, Wavelet Leaders Multifractal, and p-Leader Multifractal Method in Chatter Detection. Nanomanuf. Metrol..

[33] Rahimi M.H., Huynh H.N., Altintas Y. (2021). On-line chatter detection in milling with hybrid machine learning and physics-based model. CIRP J. Manuf. Sci. Technol..

[34] Cao H., Lei Y., He Z. (2013). Chatter identification in end milling process using wavelet packets and Hilbert–Huang transform. Int. J. Mach. Tools Manuf..

[35] Zhang P., Gao D., Lu Y., Kong L., Ma Z. (2022). Online chatter detection in milling process based on fast iterative VMD and energy ratio difference. Measurement.

[36] Chen Z., Li Z., Niu J., Zhu L. (2020). Chatter detection in milling processes using frequency-domain Rényi entropy. Int. J. Adv. Manuf. Technol..

[37] Matthew D.E., Cao H., Shi J. (2024). Advancing chatter detection: Harnessing the strength of wavelet synchrosqueezing transform and Hilbert-Huang transform techniques. J. Manuf. Process..

[38] Zhu L., Liu C., Ju C., Guo M. (2020). Vibration recognition for peripheral milling thin-walled workpieces using sample entropy and energy entropy. Int. J. Adv. Manuf. Technol..

[39] Hao Y., Zhu L., Yan B., Qin S., Cui D., Lu H. (2022). Milling chatter detection with WPD and power entropy for Ti-6Al-4V thin-walled parts based on multi-source signals fusion. Mech. Syst. Signal Process..

[40] Shannon C.E. (1948). A mathematical theory of communication. Bell Syst. Tech. J..

[41] Zhuo R., Deng Z., Li Y., Liu T. (2024). An online chatter detection and recognition method for camshaft non-circular contour high-speed grinding based on improved LMD and GAPSO-ABC-SVM. Mech. Syst. Signal Process..

[42] Rényi A. (1961). On Measures of Entropy and Information. Proceedings of the Fourth Berkeley Symposium on Mathematical Statistics and Probability, Volume 1: Contributions to the Theory of Statistics.

[43] Wei L., Wang D., Wang Y. (2025). Generalized relative entropy: New look at Rényi entropy and its exploration from complexity measures to sparsity measures with applications in machine condition monitoring. Mech. Syst. Signal Process..

[44] Florindo J.B. (2023). Renyi entropy analysis of a deep convolutional representation for texture recognition. Appl. Soft Comput..

[45] Zhang P., Gao D., Hong D., Lu Y., Wu Q., Zan S., Liao Z. (2023). Improving generalisation and accuracy of on-line milling chatter detection via a novel hybrid deep convolutional neural network. Mech. Syst. Signal Process..

[46] Wang Y., Zhang M., Tang X., Peng F., Yan R. (2022). A kMap optimized VMD-SVM model for milling chatter detection with an industrial robot. J. Intell. Manuf..

[47] Tran M.-Q., Liu M.-K., Elsisi M. (2022). Effective multi-sensor data fusion for chatter detection in milling process. ISA Trans..

[48] Sun L., Huang X., Zhao J., Wang X., Ma M. (2025). An intelligent chatter detection method for high-speed milling under variable tool-workpiece systems and cutting parameters. Mech. Syst. Signal Process..

[49] Bai Q., Wang P., Cheng K., Zhao L., Zhang Y. (2024). Machining dynamics and chatters in micro-milling: A critical review on the state-of-the-art and future perspectives. Chin. J. Aeronaut..

[50] Sun Z., Liu P., Wang Z. (2017). Real-time fault diagnosis method of battery system based on shannon entropy. Energy Procedia.

[51] Bai H., Zhao Y., Shen W. (2013). Radar radiation source identification based on the Rényi entropy feature of time-frequency distribution. J. Circuits Syst..

[52] Kim C.-J., Mayor J.R., Ni J. (2005). A static model of chip formation in microscale milling. J. Manuf. Sci. Eng..

[53] Filiz S., Conley C.M., Wasserman M.B., Ozdoganlar O.B. (2007). An experimental investigation of micro-machinability of copper 101 using tungsten carbide micro-endmills. Int. J. Mach. Tools Manuf..

